# Indications for surgical fixation of low-energy pelvic ring fractures in elderly: a systematic review

**DOI:** 10.1007/s00402-022-04438-w

**Published:** 2022-04-25

**Authors:** R. A. Timmer, S. M. Verhage, P. Krijnen, S. A. G. Meylaerts, I. B. Schipper

**Affiliations:** 1 Department of Trauma Surgery, Leiden University Medical Center, Leiden, The Netherlands; 2 Present Address: Department of Trauma Surgery, Haaglanden Medical Center, The Hague, The Netherlands; 3 Department of Trauma Surgery, Haaglanden Medical Center, The Hague, The Netherlands

**Keywords:** Pelvic fractures, Geriatric, Fragility, Osteoporosis, Surgical fixation, Indication

## Abstract

**Introduction:**

There are no generally accepted criteria for when and how to fixate osteoporotic pelvic ring fractures in elderly. This systemic review aims to summarize the currently available literature regarding the indications and methods for surgical fixation of fragility fractures of the pelvic ring in elderly patients after low-energy trauma.

**Materials and methods:**

The Pubmed and Embase databases were searched using the key words pelvic fractures, geriatric, fragility, osteoporosis, and surgical fixation, and their synonyms. Extracted data including the indication, method of operative fixation, and post-operative outcomes (pain levels, mobility, complications and mortality) were analyzed using descriptive statistics. The studies were too heterogeneous to perform a meta-analysis.

**Results:**

Eleven cohort studies (3 comparative and 8 noncomparative) were included. The methodological quality was poor to moderate; the studies were heterogeneous regarding study design and reported outcomes. In all included studies operative treatment for all fracture types was preceded by a period of conservative treatment comprising physiotherapy-guided full weight-bearing. Time to surgery differed widely. For posterior ring fixation, the majority of the included studies used minimally invasive surgery with trans-iliosacral screws. Five studies described a form of additional fixation of the anterior pelvic ring but did not report the indications.

**Conclusions:**

Fixation of low-energy pelvic ring fractures in elderly is commonly performed after a period of conservative treatment, with persistent pain as the most frequent indication for fixation. Fracture classification based on stability seems to be of secondary importance. Timing for surgical fixation of the pelvic ring fracture in elderly patients remains diverse. Large well-designed comparative prospective studies and randomized controlled trials are needed to provide clearly substantiated guidelines.

## Introduction

The incidence of osteoporotic pelvic ring fractures is increasing due to the ageing population [1, 2]. In contrast to younger patients, pelvic ring fractures in elderly are often the result of a low-energy fall and are rarely associated with hemodynamic instability or severe injuries to the pelvic organs or the surrounding soft tissue [1, 3, 4]. A growing number of studies regarding fracture characteristics, classifications and treatment algorithms for osteoporotic pelvic ring fractures are being published [5–8]. However, indications for when to perform operative fixation in this frail patient group that is susceptible for peri-operative and post-operative complications, are not clearly defined, remain controversial and are merely based on expert opinion [5, 9–13].

Routine CT-scan evaluation reveals that in up to 80% of the elderly patients an anterior pelvic ring fracture is accompanied by a posterior fracture in the pelvic ring [7, 14]. Combined anterior and posterior pelvic ring fractures may be considered (partially) unstable and tend to be associated with higher pain levels that may inhibit early mobilization [15, 16]. Since early mobilization and weight-bearing are crucial in this population, surgical fracture fixation may outweigh the potential risk associated with operative treatment [15]. Still, the majority of patients with osteoporotic pelvic ring fractures are treated non-operatively, with mobilization guided by pain levels and adequate analgesia. The development of better peri-operative imaging and the availability of minimally invasive fixation techniques have contributed to a more positive attitude towards operative treatment of elderly patients with a low-energy pelvic ring fracture. Selected patients, especially those who suffer from persistent pain or unstable fractures, may benefit substantially from surgical stabilization of the pelvic ring, to gain pain reduction, and facilitate early weight-bearing. Scientific substantiation for this suggestion is however limited and scattered.

This systemic review aims to summarize the currently available literature regarding the indications and methods for surgical fixation of fragility fractures of the pelvic ring (FFP) in elderly patients after low-energy trauma.

### Methods

A systematic review of the current literature was conducted according to the Preferred Reporting Items of Systematic reviews and Meta-Analysis (PRISMA) statement [17].

#### Search strategy

The search terms for searching the electronic databases PubMed and Embase were composed in close collaboration with a trained librarian and included the following keywords and their synonyms: pelvic fractures, geriatric, fragility, osteoporosis, and surgical fixation. The search strategy is presented in *appendix 1.*

#### Study selection

The study selection was performed independently by two authors (RT, SV). The title and abstract of the identified studies were screened using the following criteria. (1) Clinical studies, (2) including elderly patients (age > 65 yers.) suffering from a fragility fracture of the pelvic ring (3) who underwent surgical fixation of the pelvic ring and (4) published in English or Dutch were considered for inclusion in this review. The full-text papers of the potentially eligible studies were read and selected for the review if they met the same criteria and if the following information was reported: (5) type of surgical fixation, (6) the indication for surgical fixation and (7) post-operative outcomes (pain scores, mobility, complications, and/or mortality).

Additionally, the reference lists in the selected articles were screened for any relevant studies that were missed in the search.

#### Data extraction and reporting

The following study characteristics were extracted from the selected full-text papers: author, year of publication, country, study design, number of patients, mean age, gender, fracture type and mean duration of follow-up (mean and SD or median and range). The type of surgical fixation and the indication for operative fixation were extracted as well as the following patient outcomes: pain levels after surgery, mobility after surgery, mortality and complication rates. Extracted data were presented using descriptive statistics. No meta-analysis was performed for outcome data, since the studies were too heterogeneous.

#### Assessment of risk of bias

The risk of bias in the selected studies was independently assessed by two authors (RT and SV) using the Methodological Index for Non-Randomized Studies (MINORS) criteria. For non-comparative studies, this tool includes eight methodological aspects that are scored as 0 (not reported), 1 (reported but inadequate) or 2 (reported and adequate), with a maximum score of 16. For comparative studies, the tool includes 4 additional criteria (maximum score 24) [18].

## Results

**Fig. 1:**
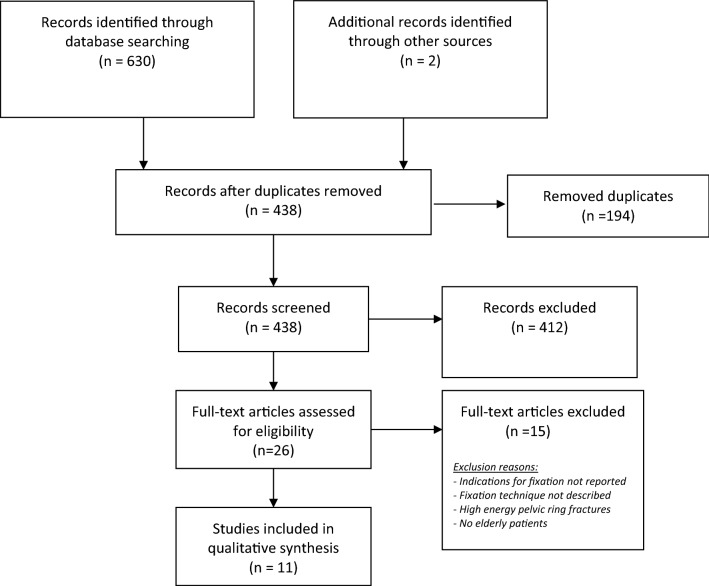
PRISMA 2009 flow diagram

The literature search resulted in 438 potentially relevant studies. Twenty-six studies were selected for full-text screening. Eleven studies met the inclusion criteria and were included in this review. The process of study selection is displayed in Fig. 1*.*

Three retrospective cohort studies had a comparative design. One of these compared the outcomes of a non-surgical and a surgical treatment group, another compared non-surgically treated patients with a mixed group of conservatively and operatively treated patients, and one study particularly focused on comparing the outcomes of FFP I/II (stable) versus FFP III/IV (unstable) fracture types after either non-surgical or surgical treatment (Table 1) [19–21]. The other eight studies were non-comparative cohort studies, addressing the indications for and outcomes after either isolated posterior fixation (one prospective and two retrospective cohort studies; Table 2) [22–24] or a combined anterior and posterior fixation (five retrospective cohort studies; Table 3) [25–29]. The mean age of the included patients per study ranged from 70 to 84 years. Mean follow-up varied between 4 weeks and 62 months.

**Table 1:** Comparative cohort studies: characteristics and outcomes

Study details	Study design	Patients, N	Mean Age (SD/range)	Female %	Fracture type, N (%)	Indications for OM/ Time to surgery	Anterior type of fixation N	Posterior Type of fixation	Mean follow-up (SD/range)	Outcomes			
										Pain	Mobility	Complications	Mortality
Hotta 2021, Japan [19]	RC FFP I-II vs FFPIII- IV	84 OM: 8 *- FFP I/II: 6* *- FFPIII/IV:2* NOM: 76 - *FFP I/II: 47* - *FFPIII/IV:29*	83.5 (7.8)	92%	FFP I: 18 FFP II: 35 FFP III: 8 FFP IV:23	Failure of NOM: Not able to stand after 10 days Surgery after: Mean 11.6 days	None	TISS	408.7 days (254)	**NR**	Change in Graham scale: FFP I-II 0.25 FFP III-IV 0.23 *p* = *0.889*	Surgical: none	**NR**
Osterhoff 2019, Germany [20]	RC Group 1 vs Group 2*	230 Group 1: OM: 60/148 NOM: 88/148 Group 2: NOM: 82	81 (60- 98)	NOM-OM: 81% NOM: 89%	**NR**	Failure of NOM: inability to mobilize after 3–5 days Anterior fixation: if displaced > 1 shaft width + pain	Plate: 8 RS: 5 INFIX: 4	TISS	Group 1: 69 months (60–85) Group 2: 44 months (41–53)	**NR**	Majeed score - Group 1: 66.1 (SD 12.6) - Group 2: 65.7 (SD 12.5) *p* = *0.910*	In hospital -Group 1 34.5% - Group 2 17.1% *p* = *0.008*	1-year: - Group 1: 34 (23%) - Group 2: 14 (17%) *p* = *0.29*
Walker 2018, Canada [21]	RC OM vs NOM	41 OM: 16 NOM: 25	OM: 78.1 NOM: 77.7	OM: 87.5% NOM: 88%	OM: LC-1: 8 Sacral U-type: 8 NOM: LC-1: 18 Sacral U-type: 7	Failure of NOM: unable to ambulate or severe posterior pelvic pain with ambulation	None	TISS	**NR**	↓VAS (pre vs. post): - OM: 3.9 - NOM:0.6 *p* < *0.01*	Ambulatory at discharge: - OM: 100% - NOM:72% *p* < *0.03*	None	None

^*^Group 1: initial NOM followed by OM if NOM failed; Group 2: only NOM

*RC* retrospective cohort, *SD* Standard deviation, *FFP* fragility fracture of the pelvis, *OM* operative management, *NOM* non-operative management, *A/P* anterior posterior, *NR* not reported, *RS* Ramus screws, *INFIX*  anterior subcutaneous internal fixation, *TISS* Transiliosacral screws

**Table 2:** Cohort studies with isolated posterior fixation: characteristics and outcomes

Study details	Study design	Patients N	Mean Age (SD/range)	Female %	Fracture type N, (%)	Indications for OM/ Time to surgery	Posterior Type of fixation	Mean follow-up (SD/range)	Outcomes			
									Pain	Mobility	Complications	Mortality
Schmerwitz 2020 Germany [22]	RC	53	79.1 (7.8)	90%	FPP II: 13 FFP III: 22 FFP IV 18	Unstable fractures (FFP III/IV) or FFP II + persistent pain Surgery after: 55.5 days (3–720)	Plate	31.5 months (6–90)	Post hospital VAS: 2.4	IOWA Pelvic Score 85.6 (55–99)	- Surgical: 7 (13%) - Post-op: 9 (17%)	1 pt. died after 24 days post-op of pneumonia
Obid 2020 Germany [23]	PC	13	83.9 (6.3)	100%	FFP II: 10 FFP III: 1 FFP IV: 2	Failure of NOM Surgery after: 2 weeks	Minimally invasive lumbo-pelvic fixation	4 weeks	NRS pre-op 7.18 (0.98) post-op 2.45 (0.93) *P* < 0.001	TMT pre-op 4.15 (SD 3.67) TMT post-op 16.39 (SD 4.61) *P* < 0.001	Surgical: 2 (15.4%) wound problem 1 rod dislocation 1 pneumonia 2	**NR**
Noser 2018 Switzerland [24]	RC	60	79.0 (9.0)	88%	FFP II: 17 FFP III: 26 FFP IV: 17	Failure of NOM Surgery after*:* 5 days	TISS 8.3% open reduction	62 months (22) 46 lost to follow-up	**NR**	Majeed score *N* = 14 available final FU: mean 65 points (SD 11) - FFP II: 67 - FFP III: 69 - FFP IV: 60 *(p* = *0.21)* 25% of all patients could return home FFP II: 6 (35%) FFP III: 5 (19%) FFP IV: 4 (24%)	Total: 26 (43.3%) - re-operation:2. (3.3%) - HA infections: 19 (31.7%) - TE 1 (1.7%) - Delirium: 7 (11.7%)	In-hospital: 2 (3.3%) 1-year: 17 (28.3%)

*RC* retrospective cohort, *SD* Standard deviation, *FFP* fragility fracture of the pelvis, *OM* operative management, *NOM* non-operative management, *A/P* anterior posterior, *NR* not reported, *TISS* transiliosacral screws, *TMT* Tinetti Mobility Test, *HA* Hospital acquired, *TE* Thrombo embolic event; *NRS*: numerical rating scale

**Table 3:** Cohort studies with combined anterior and posterior fixation: characteristics and outcomes

Study details	Study design	Patients N	Mean age (SD/range)	Female %	Fracture type N (%)	Indications for OM/Time to surgery	Anterior type of fixation N	Posterior type of fixation	Mean follow-up (SD/range)	Outcomes			
										Pain	Mobility	Complications	Mortality
Ferry 2020, USA [25]	RC	50 FFP: 32	FFP: 78.0 (9.1)	FFP: 65.6%	Sacral (Denis) Zone 1: 3.8% Zone 2: 9.4% Zone3: 6.3% Sacral U-type:31.3% LC-II: 9.4%	Failure of NOM Surgery after: 5.6 days (SD 9.4)	If surgically needed not further specified	TISS	FFP: 18.0 weeks (19)	**NR**	Ambulant post- discharge: 94% Time to ambulatory: 18.0 weeks	**NR**	1-year: 4 (12.5%)
Wong 2019, Hong Kong [26]	RC	17	80.1 (8.2)	94%	AO B1: 2 B2: 10 B3: 2 A/P column: 2 No class: 1	Failure of NOM Surgery after: 8.6 days (SD 2.4)	Anterior column screw: 17	TISS: 12	18.7 months (2.8)	Post-op ↓VAS mean 3.3 *P* < 0.001	5 pt. walked unaided 7 pt. required walking aids	Infected pin: 1 UTI: 3 acute delirium: 2 GI bleeding: 1 non-union: 1	None
Eckardt 2017 Switzerland [27]	RC	50	79.1 (8.4)	86%	FFP II: 15 FFPIII: 10 FFP IV: 25	Failure of NOM	Plate: 9 EF: 5	TISS	805 days (453)	VAS = 0, *n* = 20	**NR**	I-O bleeding: 1 Malpositioning screw:1 Screw loosening + re-op: 9 (18%)	1-year: 5 (10%)
Schmitz 2015 Germany [28]	RC	15	79.9 (9.0)	93%	FFP II: 5 FFP III: 1 FFP IV: 9	Failure of NOM Surgery after: 32 days (SD 27)	EF: 5 Supra acetabular screw: 3	caTIFI: 4 TIFI: 4 caIF: 7	**NR**	**NR**	**NR**	Re-operations: 0 Screws hit iliosacral joint: 4 Screw perforated Ilium cortex: 1 Cement leakage: 0	**NR**
Studer 2013 Switzerland [29]	RC	132 OM: 5	84 (66–100)	86%	Pubic rami fractures	Failure of NOM Surgery after: 4–6 weeks	Plate: 2	TISS	1 year 2 (1.5%) Lost FU	**NR**	**NR**	**NR**	1-year: 24 (18.5%)

*RC* retrospective cohort, *SD* Standard deviation, *FFP* fragility fracture of the pelvis, *OM* operative management, *NOM* non-operative management; *A/P*: anterior posterior, *UTI* urinary tract infection, *I-O* intra-operative, *GI* Gastro-intestinal, *EF* External fixator, *TISS* transiliosacral screws, *caTIFI* cement augmented trans-iliac internal fixator, *caIF* cement augmented internal fixator, *NR* not reported, *N/A* not applicable

### Methodological quality

According to the MINORS criteria the methodological quality of the selected studies was low to moderate (Table 4). The MINORS scores for the three included comparative studies ranged between 13 and 17 and for the remaining non-comparative studies between 5–11. All except one study had a retrospective study design. None of the studies reported a sample size calculation and whether the endpoint assessment was unbiased.

**Table 4:** MINORS criteria included studies

Study	A clearly stated aim	Inclusion of consecutive patients	Prospective collection of data	Endpoint appropriate to the aim of the study	Unbiased assessment of the study endpoint	Follow-up period appropriate to the aim of the study	Loss of follow-up less than 5%	Prospective calculation of the study size	Additional criteria for comparative studies				Total
									An adequate control group	Contemporary group	Baseline equivalent of groups	Adequate statistical analysis	
Hotta et al. [19]	2	2	1	2	0	2	2	0	0	0	2	2	**15**
Osterhoff et al. [20]	2	2	1	2	0	2	1	0	1	2	2	2	**17**
Walker et al. [21]	2	1	1	2	0	0	1	0	1	1	2	2	**13**
Schmertiz et al. [22]	2	0	0	2	0	2	1	0	N/A	N/A	N/A	N/A	**7**
Obid et al. [23]	2	2	2	2	0	1	0	0	N/A	N/A	N/A	N/A	**9**
Noser et al. [24]	2	2	1	2	0	2	1	0	N/A	N/A	N/A	N/A	**10**
Ferry et al. [25]	2	1	1	2	0	2	1	0	N/A	N/A	N/A	N/A	**9**
Wong et al. [26]	2	2	1	2	0	2	2	0	N/A	N/A	N/A	N/A	**11**
Eckhartd et al. [27]	2	2	1	2	0	2	1	0	N/A	N/A	N/A	N/A	**10**
Schmitz. et al. [28]	2	0	1	1	0	2	0	0	N/A	N/A	N/A	N/A	**6**
Studer et al. [29]	2	2	1	2	0	2	2	0	N/A	N/A	N/A	N/A	**11**

0 = not reported; 1 = reported but inadequate; 2 = reported and adequate; N/A: not applicable

### Comparative cohort studies

Three comparative retrospective cohort studies were included in this review (Table 1). Osterhoff et al. compared two groups with a mean age of 81 years (range 60–98): group 1 received non-operative treatment only (*n* = 82) and group 2 received non-operative treatment followed by operative treatment if the patient was unable to mobilize after 3–5 days (*n* = 148, of which 60 received operative treatment and 88 did not) [20]. Surgical fixation in group 2 was performed using trans-iliosacral screw fixation. Majeed mobility scores and the one-year mortality rates did not differ between the two groups. In-hospital complications occurred significantly more often in group 2.

The study by Walker et al. included 41 patients with isolated sacral fractures and a mean age of 78 years in both groups [21]. They excluded patients with an absolute operative indication because of an unstable fracture. All included patients were treated conservatively for an unspecified period of time and the indication for operation was set if patients were unable to ambulate or were experiencing severe pain during mobilization. At hospital discharge the 16 operatively treated patients had significantly more reduced pain scores compared to the conservatively treated group of 25 patients (operative: − 3.9 points on scale 0–10; non-operative: − 0.6 points; *p* < 0.01). All patients who underwent surgical fixation were ambulatory at discharge compared to 72% of the conservatively treated patients (*p* < 0.03).

Hotta et al. compared patients suffering from stable (FFP I/II, *n* = 53) versus unstable pelvic ring fractures (FFP III/IV, *n* = 31) with mean age of 84 years [19]. Primarily, all patients were treated non-operatively. If patients were not able to ambulate after 10 days of conservative treatment, comprising physiotherapy-guided full weight-bearing and adequate analgesics, fixation of the posterior pelvic ring was indicated, and performed using iliosacral and/or trans-sacral screws. Eight patients (FFP I/II: 6; FFP III/IV:2) underwent surgical fixation, the remaining 76 patients (FFP I/II: 47; FFP III/IV: 29) did not. The change in functional outcomes according to the Graham scale did not differ between the FFP I-II and FFP III-IV groups (FFP I-II: 0.25 vs. FFP III-IV: 0.23 *p* = 0.89). Functional outcomes after surgical and non-surgical regimes were not reported or compared. No complications occurred after surgical fixation.

### Non-comparative studies

All eight non-comparative studies reported ‘failure of conservative treatment’ as the indication to perform a surgical fixation of the pelvic ring (Tables 2, 3). Failure of conservative treatment was defined as the patient not being able to mobilize due to persistent pain. The mean time until failure of conservative treatment differed between these studies and ranged from 3 to 241 days after trauma (Table 5). Schmerwitz et al. followed a slightly different indication for operative treatment. They specifically described the presence of an unstable fracture (FFP III/IV) or FFP II type in combination with persistent pain after conservative treatment (including full weight-bearing if possible) as an indication to perform surgical fixation of the pelvic ring fracture. (Table 5) [22].

**Table 5:** Indications for operative management (OM) and definition of non-operative management (NOM)

Study details	Indication for OM	Definition of NOM	Definition failure of NOM	Predefined period of NOM
Hotta 2021, Japan [19]	Failure of NOM	Full weight-bearing exercises within pain limits 40 min. of physiotherapy a day + adequate analgesics	Difficulty of standing: difficulty with auxiliary standing on 1 leg and /or 2 legs due to permanent pain	10 days
Osterhoff 2019, Germany [20]	Failure of NOM	Physiotherapy-guided full weight-bearing + adequate analgesics	Patient was not able to ambulate with a walker or crutches	3–5 days
Walker 2018, Canada [21]	Failure of NOM	Physiotherapy-guided full weight-bearing + adequate analgesics	unable to ambulate or severe posterior pelvic pain with ambulation	*NR*
Schmerwitz 2020 Germany [22]	Unstable fractures (FFP III/IV) or FFP II after failure NOM	Physiotherapy-guided full weight-bearing + adequate analgesics	Patient was not able to ambulate	*NR*
Obid 2020 Germany [23]	Failure of NOM	Physiotherapy-guided full weight-bearing + adequate analgesics	Patient were bedridden due to pain and were ambulatory before fractures	2 weeks
Noser 2018 Switzerland [24]	Failure NOM No contra-indications against general anesthesia	Physiotherapy-guided full weight-bearing + adequate analgesics	Not able to mobilize on walking aids	5 days
Ferry 2020, USA [25]	Failure of NOM	*not further specified*	Patient was not able to ambulate	3–5 days
Wong 2019, Hong Kong [26]	Failure of NOM	Physical therapy-guided full weight-bearing using adequate analgesics	Impaired mobilization due to persistent pain	*NR*
Eckhard 2017 Switzerland [27]	Failure of NOM	*not further specified*	Persistent pain limiting mobilization	*NR*
Schmitz 2015 Germany [28]	Displaced fractures or Failure NOM	Physical therapy-guided full weight-bearing using adequate analgesics	Not able to mobilize out of bed	*NR*
Studer 2013 Switzerland [29]	Failure of NOM	*not further specified*	Persistent pain limiting mobilization	4–6 weeks

*OM* operative management, *NOM* non-operative management, *FFP* fragility fracture of the pelvis, *NR* not reported

### Method of posterior ring fixation

For posterior ring fixation, the majority of the included studies used minimally invasive surgery with trans-iliosacral screws [20, 21, 24–29].

Complication rates ranged from 0 to 46%. Hospital-acquired infections (e.g. urinary tract or pulmonary infection) were most often reported with rates up to 34%. Surgical site infections and secondary screw loosening were seen less often, with rates up to 13% and 18%, respectively. The 1-year mortality rate ranged from 12.5 to 18.5% (Tables 1, 2, 3).

One of the included studies performed a posterior fixation combined with cement augmentation to prevent secondary screw dislocation. Schmitz et al. used a cement-augmented transiliac internal fixator (caTIFI) [28]. All screws were placed using intra-operative fluoroscopy. No cement leakage was reported, but in five of the 15 included patients, a malposition of screws was documented on the post-operative CT-scan. None of the patients received a second operation. Follow-up on pain and mobility scores were not reported (Table 3) [28].

Schmerwitz et al. performed a minimally invasive locking compression plate fixation of the posterior pelvic ring in 53 patients suffering from FP III/IV or a FFP II type pelvic fractures in combination with persistent pain, after a period of conservative treatment [22]. Complications directly related to the surgery were reported in 13% of the patients. Pain levels and IOWA pelvic ring scores (including mobility levels and daily activities) upon hospital discharge were found satisfying. (Table 2) Obid et al. described the results after fixation of the posterior pelvic ring using minimally invasive lumbopelvic fixation in 13 patients [23]. All patients were operated on after failure of conservative treatment, after a duration of 2 weeks on average. Surgical complications were reported in 15% of the patients. Pain and mobility scores after surgery were significantly improved when compared to pre-operative levels (Table 2).

#### Method of anterior ring fixation

Six of the included studies performed additional fixation of the anterior pelvic ring, using different techniques [20, 25–29]. Plate fixation of the symphysis or of the rami only, was described in three studies [20, 27, 29]. Trans-pubic or supra-acetabular screw fixation was performed in three studies and external fixators were placed in two studies [20, 26–28]. Ferry et al. did perform additional anterior fixation in their study, however without describing the used technique or approach [25]. The indication to perform anterior fixation in addition to posterior fixation, was only described in one of the included studies. The comparative study by Osterhoff et al. performed anterior plate fixation or an anterior subcutaneous internal fixator (INFIX) if patients suffered from a displaced fracture of the ramus superior/inferior (> 1 shaft width) and persistent pain over the anterior pelvic ring [20].

## Discussion

The purpose of this study was to systematically describe the indications for surgical fixation of pelvic ring fractures in elderly patients after a low-energy trauma.

Although slowly, the body of literature concerning when and how to operate pelvic ring fractures resulting from low-energy trauma in elderly is growing. The current review presents results that suggest a consensus about the indication for fixation of these fractures. In none of the included studies, the low-energy pelvic ring fractures were directly operated after trauma, so that surgery was always preceded by a period of conservative treatment.

In general, patients who suffered from persistent pain and for this reason were unable to mobilize after a certain period of supervised weight-bearing, were selected for surgical fixation of the pelvic ring. Duration of the conservative treatment period differed widely. Performing immediate surgical fixation can lead to overtreatment inducing unnecessary risks related to surgery. On the other hand, if surgery is postponed for a longer conservative period, the most painful period of healing has passed and some patients will be undertreated. Furthermore, none of the studies included some sort of frailty index and therefore it was not possible to assess its impact on whether to perform surgical fixation. In our opinion, this is remarkable since for this population, frailty could affect the decision whether to choose for surgical intervention. The findings of the current review are partially in line with earlier published recommendations by Rommens et al. and Oberkircher et al. who advised conservative treatment for undisplaced fractures and stable fractures (FFP I/II), and immediate surgical fixation for unstable displaced fractures (FFP III/IV) based on their extensive clinical experience [5, 8]. When conservative treatment fails, meaning that the patient is experiencing immobilizing pain, both studies recommend repeating diagnostic imaging (Fluoroscopy and CT-scan evaluation) and to consider surgical fixation. Remarkably, Hotta et al. found, in comparing the results of stable fractures (FFP I/II) and instable fractures (FFP III/IV) [19] that after all patients had been admitted to a conservative treatment period of 10 days, only 8 of the 84 patients (FFP I/II:6/53; FFP III/IV:2/31) were unable to stand and were operated on. The remaining 76 patients (FFP I/II:47/53; FFP III/IV:29/31) were treated non-operatively. At follow-up, no significant difference in mobility between the FFP I/II and FFP III/IV groups was reported. This raises the question if immediate surgical fixation of FFP III/IV fractures is warranted [5, 8].

Five studies in the current review described a form of additional fixation of the anterior pelvic ring. Only Osterhoff et al. stated a clear indication for performing this additional fixation. They performed anterior fixation of the pelvic ring simultaneously with trans-iliosacral screws if the anterior pelvic ring fracture of either the ramus superior or inferior was displaced more than one shaft width and/or the patient suffered from persistent inguinal pain [20]. Recently, Rommens et al. published two studies presenting the surgical options for anterior fixation but did not provide a clear indication for when to additionally fixate the anterior ring [13, 30].

Intra-operative stability testing using fluoroscopy may be useful to help decide whether to perform additional anterior fixation. This technique, enabling the surgeon to test the stability of the pelvic ring was found to be promising to determine the need for fixating undisplaced LC-1 type fractures in younger patients [31]. It may also be used in elderly patients, to assess the stability of the pelvic ring after the initial posterior fixation. If there is still displacement of the fracture in the anterior pelvic ring under stress using fluoroscopy indicating possible persistent ring instability, anterior fixation can be considered and performed in the same session.

However, the amount of applied force and the visual estimation of displacement during the examination on fluoroscopy are both subjective, are therefore difficult to quantify and can differ significantly between surgeons [32]. Furthermore, if anterior ring movement is observed under compression or distraction, does that mean the ring is unstable, and how much movement should be considered to reflect instability? This limitation should be taken into account when using this technique and more reproducible data on this method would be helpful before considering it a standard tool in decision making for pelvic fracture treatment.

The studies included in this review described different fixation techniques with comparable outcomes regarding post-operative infections and secondary screw loosening. The transiliac screw fixation seemed the preferred fixation technique for fractures of the posterior pelvic ring and was used in eight of the eleven included studies. The anterior pelvic ring was fixated using plate osteosynthesis in three studies and two studies used transpubic screws. The osteoporotic bone in elderly patients can be challenging in terms of achieving adequate grip with higher risk of secondary screw loosening [33]. For this reason, one of the included studies used cement-augmented posterior screw fixation after which no secondary screw loosening was reported. According to the same principle used in for example humeral head screw fixation, cement augmentation using a minimal amount of cement at the tip of iliosacral screws can help to increase the strength of anchorage and reduce the risk of secondary screw loosening [34]. Caution should be taken regarding the amount of cement used, because the use of large quantities can lead to cement leakage, with consequent adverse effects [35–37]. When fully treated transiliac–transsacral screws are placed, there is no indication for cement, since correctly placed fully threaded screws ending in the contralateral iliac bone usually have satisfactory grip. Fully threaded screws provide superior biomechanical stability and are preferred over partially threaded transiliac–transsacral or trans-iliosacral screws [38, 39].

## Conclusion

The current review shows that fixation of low-energy pelvic ring fractures in elderly is commonly performed after a period of conservative treatment, with persistent pain as the most frequent indication for fixation. Fracture classification based on stability seems to be of secondary importance. Timing and other indications for surgical fixation of the pelvic ring fracture in elderly patients remain diverse. However, these findings are mainly based on observational non-comparative retrospective cohorts. Clear indications for when, who and how to operate should be substantiated by the results of large, preferably randomized, prospective studies comparing surgical with non-surgical regimes in elderly patients suffering from an osteoporotic pelvic ring fracture.
